# The morphology and morphometry of the fetal fallopian canal: a microtomographic study

**DOI:** 10.1007/s00276-014-1395-2

**Published:** 2014-12-06

**Authors:** Tymon Skadorwa, Mateusz Maślanka, Bogdan Ciszek

**Affiliations:** 1 Department of Descriptive and Clinical Anatomy, The Medical University of Warsaw, 5 Chałubińskiego St., 02004 Warsaw, Poland; 2 Department of Pediatric Neurosurgery, Bogdanowicz Memorial Hospital for Children in Warsaw, 4/24 Nieklanska St., 03924 Warsaw, Poland

**Keywords:** Fallopian canal, Facial nerve, Microtomography, Fetal anatomy

## Abstract

**Background:**

The canal for facial nerve (the fallopian canal, FC) is a bony structure passing through the petrous part of the temporal bone. The anatomy of this demanding and important for oto- and neurosurgeons structure is well described in literature. Among several studies on radiological anatomy of this region, still little papers focus on the developmental measurements in prenatal period.

**Aim:**

Assessment of a microtomographic appearance of FC and dimensions based on available landmarks.

**Method:**

The study was performed on 22 fetal temporal bones aged 16–27 Hbd. Specimens were scanned in micro-CT scanner. Length (FC1, FC2) and width (FC1W, FC2W) of the labyrinthine and tympanic portions of FC, angle of the first curve of FC (A1-2), length of the internal acoustic meatus (IAM), distance from FC to the basal cochlear turn (BCT) and to the lateral semicircular canal (LSC) were measured.

**Results:**

The paper discusses problems and a value of micro-CT in neuroanatomical studies. FC was found in 20/22 cases. Average value of all distances measured was: FC1 1.38 ± 0.35 mm; FC2 6.68 ± 1.34 mm; FC1W 1.07 ± 0.1 mm; FC2W 1.25 ± 0.13 mm; A1-2 87.24 ± 4.05°; IAM 4.89 ± 0.60 mm; BCT 0.35 ± 0.05 mm; LSC 0.55 ± 0.05 mm.

**Conclusions:**

Labyrinthine portion starts to ossify between 16th and 18th weeks of gestation and tympanic portion is fully ossified only after 20th week. Labyrinthine and tympanic portion of FC and the IAM elongate with age, whereas the angle of the first curve of FC and the distances to the BCT and the LSC remain stable and present no correlation with age.

## Background

The canal for facial nerve (the fallopian canal, FC) is a bony structure passing through the petrous part of the temporal bone. It contains an intracranial portion of the seventh cranial nerve. The canal is in close anatomical relation with inner and middle ear structures; therefore, it is usually divided into three sections: (1) labyrinthine—originating close to the bony labyrinth; (2) tympanic—enclosed in medial and posterior wall of the tympanic cavity; and (3) mastoid—coursing through the mastoid process and ending in the skullbase, in the stylomastoid foramen [29]. Being anatomically a complex entity, FC appears as difficult and demanding region for otosurgeons—although, its specific anatomical relationship with the bony labyrinth and tympanic cavity give opportunities to reconstruct the damage of the facial nerve [7], the intraoperative or postoperative complications may include harmful consequences, such as infectious reactions, temporary or permanent peripheral facial nerve palsy or hearing disturbances [14]. The use of modern and advanced antibiotic therapies has significantly reduced the occurrence of intracranial bacterial infections, as a complication of otitis. However, the risk of involvement of the facial nerve in this pathology is still high and reaches up to 38 % of cases [8, 20].

The FC develops within the temporal bone complex, which starts to develop between second and third month of prenatal life [18]. The elements of the temporal bone complex, structures of the inner and middle ear, during development enclose the structures originating from pharyngeal arches, such as facial nerve [2, 13]. The facial nerve canal starts to ossify along with the otic capsule in approximately 15th week of gestation and develops throughout pregnancy, but is still not totally enclosed after birth [3]. The process of ossification promotes anatomical structures to be visualized and diagnosed by conventional radiography, magnetic resonance imaging (MRI) techniques, classical computed tomography or high-resolution computed tomography (HRCT), which is nowadays the most commonly used method to visualize the petrous part of the temporal bone [17, 18, 22]. None of these methods, however, is able to visualize internal structures of the pyramid as precisely as computed microtomography, which so far has mostly been used in research, especially in stomatology and maxillary surgery [28] and in the experimental assessment of the temporal bone [21].

First anatomical description of the facial nerve canal was given by G. Falloppio in the sixteenth century [11]. After its recognition, FC was described throughout decades from distinct perspectives. Most of the specifications appeared along with the studies on the temporal bone and its internal architecture [6]. Apart from them, however, the region of FC has often been mentioned in neurological considerations on the symptoms of facial nerve diseases [1, 16]. Developmental depictions gave a further insight into the formation of FC and its surroundings [13]. These considered histological studies and measurements on fetal specimens [5]. Finally, practical anatomy of the facial nerve canal was given by surgical and radiological descriptions [3, 4]. Modern studies have shown the significance of understanding of developmental anatomy in terms of clinical implications, including up-to-date diagnostic imaging and surgical treatment.

## Aim

Numerous papers on FC describe its sections, contents and topographical features. Among several studies on radiological anatomy of this region, still little papers focus on the developmental measurements in prenatal period. Therefore, the aim of our study includes microtomographic appearance of the fetal facial nerve canal with dimensions based on available landmarks. We also aimed to determine the usefulness of micro-CT pictures in visualization of the fetal fallopian canal and basing on them to assess the regularity of FC in fetuses aged from 16 to 27 Hbd.

## Materials and methods

The study was performed on 11 fetal specimens (22 temporal bones) from the collection of Department of Descriptive and Clinical Anatomy, Medical University of Warsaw, aged from 16 to 27 Hbd, both sexes (5 females, 6 males), without congenital malformations (basing on karyotype and their medical history). In the first phase, fetal head specimens were scanned with micro-CT scanner (SkyScan 1076; slice thickness 35 µm; 100 kV X-ray source; 11 Mp camera with resolution up to 8000 × 8000 pixels in every slice; down to 9 µm in vivo 3D spatial resolution; the exposition performed with source voltage of 80 kV and current of 124 μA; with 0.025 mm Titanium Filter). Scans obtained in micro-CT scanner allowed to analyze the anatomy of FC from distinct aspects, from which horizontal and sagittal planes were found the most convenient.

In the next phase, all scans were analyzed in Ginkgo CADx 3.3.0 software and obtained data were analyzed statistically with the use of StatSoft Statistica 10 software.

**Fig. 1:**
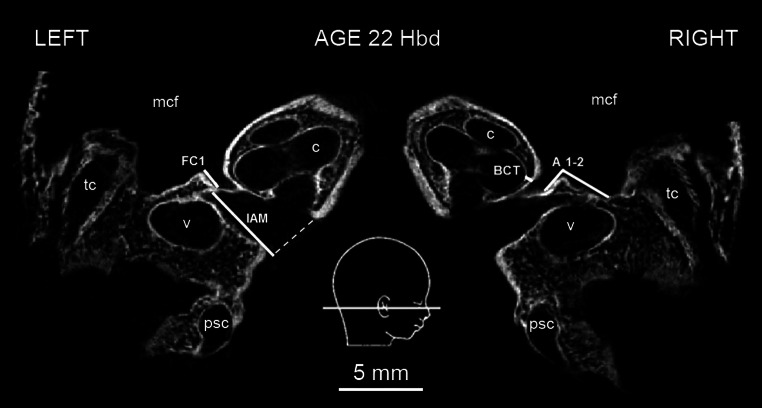
Micro-CT scan of the fetal temporal bones at 22 Hbd with measured dimensions (horizontal section). *BCT* basal cochlear turn, *c* cochlea, *mcf* middle cranial fossa, *psc* posterior semicircular canal, *tc* tympanic cavity, *v* vestibule. *A1-2*, *FC1*, *IAM—*parameters described in the text and Table 1

**Fig. 2:**
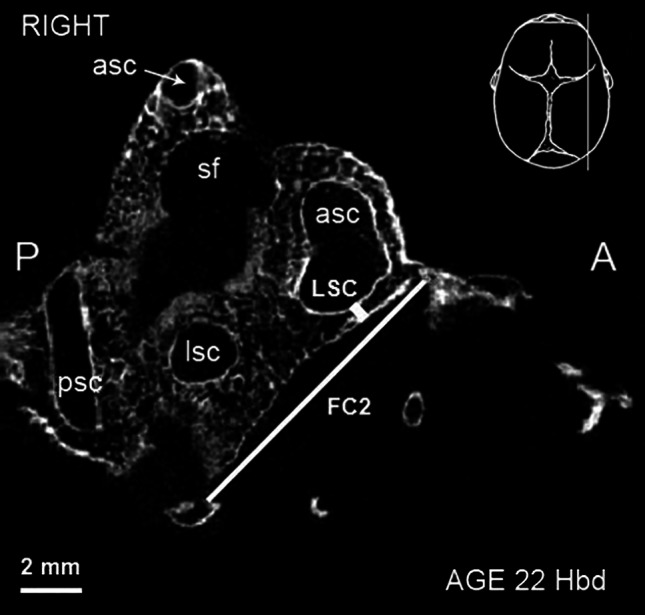
Micro-CT scan of the right fetal temporal bone at 22 Hbd (sagittal section). *asc* anterior semicircular canal, *lsc* lateral semicircular canal, *psc* posterior semicircular canal, *sf* subarcuate fossa. *FC2*, *LSC—*parameters described in the text and Table 1

**Fig. 3:**
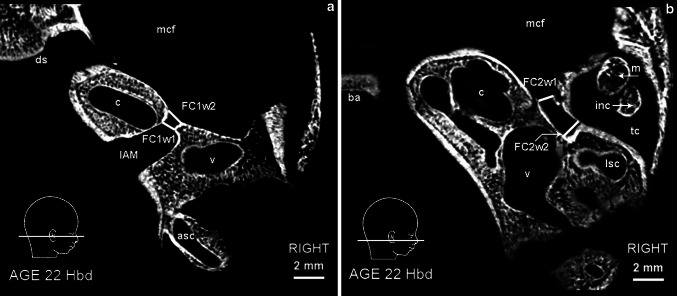
Micro-CT scan of the right temporal bone at 22 Hbd (horizontal section). **a** *asc* anterior semicircular canal, *c* cochlea, *ds* dorsum sellae, *mcf* middle cranial fossa, *v* vestibule. *FC1w1*, *FC1w2*, *IAM*—parameters described in the text and Table 1. **b** *ba* basilar portion of the occipital bone, *c* cochlea, *inc* incus, *m* malleus, *mcf* middle cranial fossa, *lsc* lateral semicircular canal, *tc* tympanic cavity, *v* vestibule. *FC2w1*, *FC2w2—*parameters described in the text and Table 1

To describe morphology of FC, several parameters were measured in horizontal and sagittal planes. These included: (1) length of the labyrinthine portion of FC (FC1, Fig. 1; Table 1); (2) angle of the first curve of FC (A1-2, Fig. 1; Table 1); (3) length of the tympanic portion of FC (FC2, Fig. 2; Table 1); (4) width of the labyrinthine portion of FC (FC1W, Fig. 3a; Table 1) and (5) width of the tympanic portion of FC (FC2W, Fig. 3b; Table 1).

**Table 1:** Description of parameters used in the study with the measurement plane

Parameter	Description	Plane
FC1	Distance between fundus of the internal acoustic meatus and the most anterior point of posterior wall of FC^a^	Horizontal
A1-2	Angle of the first curve of FC	Horizontal
FC2	Distance between the most anterior and the most posterior point of posterior wall of FC	Sagittal
FC1W	Calculated as an average from 2 auxiliary parameters (FC1w1 and FC1w2) measured between the bony walls of FC at the proximal and distal region of the labyrinthine portion	Horizontal
FC2W	Calculated as an average from 2 auxiliary parameters (FC2w1 and FC2w2) measured between the bony walls of FC at the proximal and distal region of the tympanic portion	Horizontal
IAM	Distance between inferior part of opening of the internal acoustic meatus and fundus of the meatus	Horizontal
BCT	The shortest distance between medial wall of labyrinthine portion of FC and the basal cochlear turn	Horizontal
LSC	The shortest distance between posterior wall of tympanic portion of FC and the lateral semicircular canal	Sagittal
BD	The greatest distance measured between external tables of both temporal squamae at the level of fundus of IAM	Horizontal

*FC* fallopian canal, *CT* computed tomography, *IAM* internal acoustic meatus, ^a^ anterior wall from the aspect of the tympanic cavity (where the facial geniculum ends) was not visible in micro-CT scans

To complete the description of FC, distances to the most commonly used in literature typical proximity landmarks were measured: (6) length of the internal acoustic meatus (IAM, Fig. 1; Table 1); (7) the shortest distance between FC and the basal cochlear turn (referring to the lowest width of the bony lamina separating these structures, BCT, Fig. 1; Table 1)—both distances measured in horizontal plane; (8) the shortest distance between FC and the lateral semicircular canal (referring to the lowest width of the bony lamina separating these structures, LSC, Fig. 2; Table 1)—measured in sagittal plane. Additionally, a bitemporal diameter (referred to the distance between the lateralmost points of the skull) was measured in the horizontal plane to compare the dimensions of FC with the general size of the cranium (BD, Table 1).

## Results

**Fig. 4:**
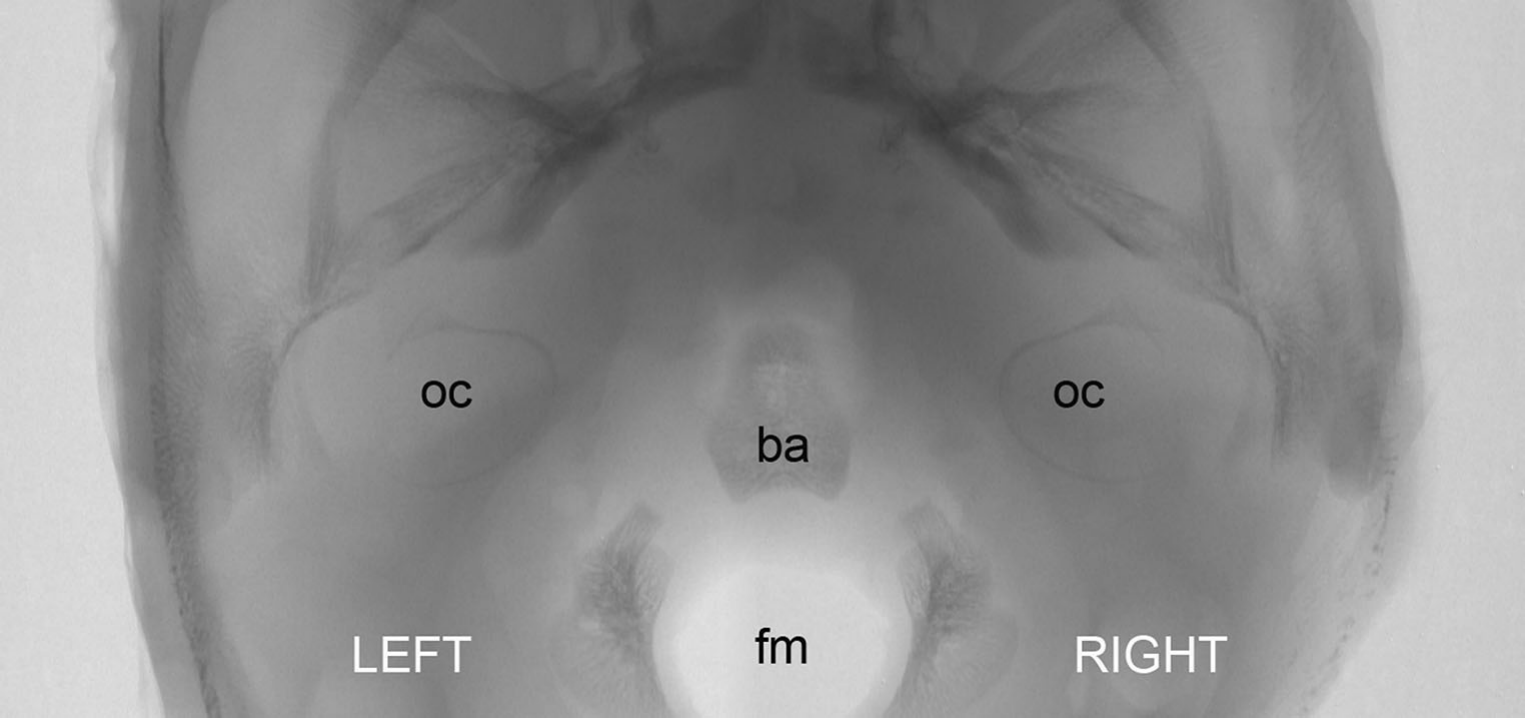
Rendered model of the fetal skullbase at 16 Hbd (horizontal aspect). *ba* basilar portion of the occipital bone, *fm* foramen magnum, *oc* otic capsule

**Fig. 5:**
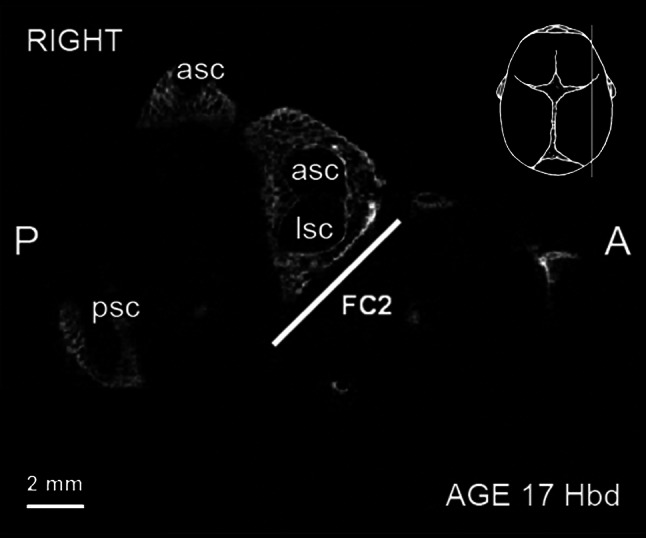
Micro-CT of the fetal temporal bone at 17 Hbd (sagittal cut). *asc* anterior semicircular canal, *lsc* lateral semicircular canal, *psc* posterior semicircular canal. *FC2—*parameter described in the text and Table 1

Microtomographic scans of head specimens showed FC in almost all examined fetuses. In one case (fetus at the age of 16 Hbd), we were unable to identify FC due to the lack of ossification of otic structures. All visualized FC were found in fetuses over 18 Hbd. Figure 4 demonstrates an early stage of ossification of otic capsules (specimen at 16 Hbd). Neither was possible to identify full length of FC2 (no appearance of second curve of the facial nerve canal) in fetuses younger than 20.5 Hbd (Fig. 5). In the remaining cases, all designed parameters were identified and measured.

FC was described basing on raw micro-CT scans in horizontal and sagittal planes. Labyrinthine portion was identified as a space between posterolateral aspect of the cochlea and anteromedial aspect of the vestibule. Its posterior wall was visualized clearly as a continuous bony table ending at the first curve of FC. Its anterior wall was not noticeable lateral to the cochlea in all cases.

**Fig. 6:**
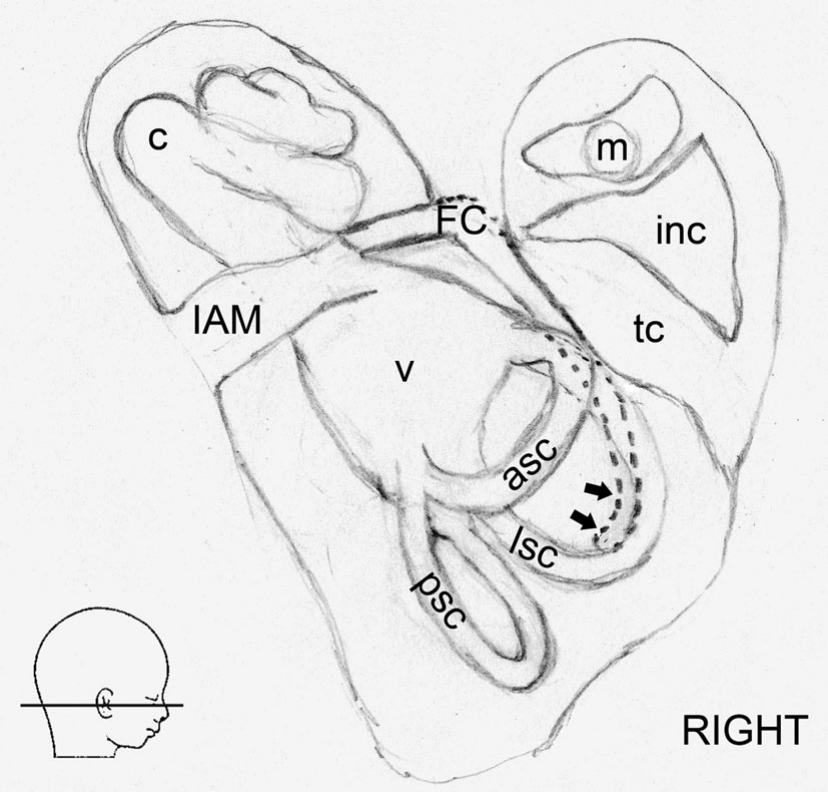
A scheme demonstrating the topography of the right fetal fallopian canal (horizontal aspect). *FC* the fallopian canal, *asc* anterior semicircular canal, *c* cochlea, *inc* incus, *lsc* lateral semicircular canal, *m* malleus, *psc* posterior semicircular canal, *tc* tympanic cavity, *v* vestibule, *IAM* internal acoustic meatus. *Arrows* indicate the course of the mastoid portion of FC

The tympanic portion was defined inferior to lateral semicircular canal and posterior to the tympanic cavity in sagittal plane. Its starting point is at the first curve of FC and endpoint in the place where FC bends for the second time. Microtomographic scans showed its posterior wall, whereas the anterior boundary of this portion could not be visualized. The boundaries of the mastoid portion of FC neither could be visualized nor measured in our material (Fig. 6).

The first curve of FC was observed between the labyrinthine and tympanic portions of FC. Posterior walls of these portions were continuous and their longitudinal axes determined the angle between them.

The width of examined portions of FC was determined by the calculation of two dimensions taken according to Table 1. The auxiliary parameters were measured between complete and continuous walls of FC in the labyrinthine and tympanic portion at the most proximal and distal points available (see Fig. 3a, b).

The IAM appeared as a space medial to the vestibule and posterior to the cochlea (see Fig. 2). It narrowed anteriorly and terminated as a blunt end closed with transverse bony plate called the fundus of IAM.

Thin bony laminae separated labyrinthine portion of FC from the basal cochlear turn and tympanic portion of FC from the lateral semicircular canal. The thickness of these laminae varied along the course of FC.

The values and min–max ranges of all direct parameters of FC are presented in Table 2. Any significant differences between right and left side were observed. Table 3 presents the values of additional parameters measured according to the literature.

**Table 2:** Descriptive statistics of direct parameters of FC

No.	Sex	Age (Hbd)	FC1 (mm)		A1-2 (°)		FC2 (mm)		FC1W (mm)		FC2W (mm)	
			R	L	R	L	R	L	R	L	R	L
1	M	16.0	ND	ND	ND	ND	ND	ND	ND	ND	ND	ND
2	F	18.0	1.30	1.40	85.00	82.00	ND	ND	1.05	1.20	1.19	1.31
3	F	20.5	1.10	1.00	90.80	88.00	ND	ND	1.20	1.11	1.38	1.56
4	F	20.5	0.80	0.70	81.00	81.00	6.40	5.80	1.13	1.19	1.19	1.19
5	M	21.0	1.10	1.10	93.00	90.00	5.80	5.20	1.07	1.00	1.13	1.10
6	M	21.0	1.50	1.60	90.00	91.00	6.10	6.50	1.18	1.03	1.10	1.13
7	F	22.0	1.70	1.80	82.00	83.00	7.20	8.00	1.04	0.94	1.20	1.36
8	M	23.5	1.80	1.60	87.90	90.00	5.10	4.50	1.06	1.04	1.32	1.27
9	M	23.5	1.40	1.20	87.00	83.40	8.00	6.90	1.22	1.20	1.38	1.37
10	M	24.0	1.30	1.30	88.10	86.50	7.00	7.40	0.92	1.01	1.11	1.12
11	F	27.0	2.00	1.80	93.00	92.00	7.00	10.00	0.89	0.96	1.41	1.29
Avg		21.5	1.40	1.35	87.78	86.69	6.58	6.79	1.07	1.07	1.24	1.27
			1.38		87.24		6.68		1.07		1.25	
SD		3.0	0.36	0.36	4.17	4.07	0.91	1.73	0.11	0.10	0.12	0.14
			0.35		4.05		1.34		0.10		0.13	
Min		16.0	0.80	0.70	81.00	81.00	5.10	4.50	0.89	0.94	1.10	1.10
			0.70		81.00		4.50		0.89		1.10	
Max		27.0	2.00	1.80	93.00	92.00	8.00	10.00	1.22	1.20	1.41	1.56
			1.80		92.00		10.00		1.22		1.56	

*avg* average value, *ND* no data

**Table 3:** Descriptive statistics of additional parameters measured according to the literature

No.	Sex	Age (Hbd)	IAM (mm)		BCT (mm)		LSC (mm)		BD (mm)
			R	L	R	R	L	R	
1	M	16.0	ND	ND	ND	ND	ND	ND	ND
2	F	18.0	4.50	4.70	0.30	4.50	4.70	0.30	38.30
3	F	20.5	3.80	4.10	0.30	3.80	4.10	0.30	42.80
4	F	20.5	5.80	5.80	0.30	5.80	5.80	0.30	42.70
5	M	21.0	4.10	4.00	0.40	4.10	4.00	0.40	43.00
6	M	21.0	5.10	5.00	0.40	5.10	5.00	0.40	43.30
7	F	22.0	5.00	5.10	0.40	5.00	5.10	0.40	45.40
8	M	23.5	4.60	4.60	0.30	4.60	4.60	0.30	45.30
9	M	23.5	5.10	5.10	0.40	5.10	5.10	0.40	45.90
10	M	24.0	5.90	5.40	0.30	5.90	5.40	0.30	46.00
11	F	27.0	4.70	5.30	0.30	4.70	5.30	0.30	51.30
Avg		21.5	4.86	4.91	0.34	4.86	4.91	0.34	44.40
			4.89		0.35		0.55		
SD		3.0	0.67	0.57	0.05	0.67	0.57	0.05	3.33
			0.60		0.05		0.05		
Min		16.0	3.80	4.00	0.30	3.80	4.00	0.30	38.30
			4.00		0.30		0.50		
Max		27.0	5.90	5.80	0.40	5.90	5.80	0.40	51.30
			5.80		0.40		0.60		

*avg* average value, *ND* no data

## Discussion

Microtomographic scanning provides an unusual insight into the complex topography of fetal FC. It gives an opportunity to analyze the exact sites of ossification of the temporal bone without dissection, as in the course of routine preparation of histological slides [2, 5, 10]. Another advantage of micro-CT is a combination of an easiness of performing with precision in projection of anatomical details. Microtomographic study also gives an opportunity to observe structures in 3D which is impossible in single X-ray photo. Unfortunately, the observation is limited to ossified structures, which implicates the possibility of visualization of the developing fallopian canal only after 18th week of gestation.

The analyzed scans show that both FC and the labyrinth start to ossify about 16th week of pregnancy and the ossification rapidly progresses in peripheral direction from the cochlea. This pattern of ossification is well described in literature [18]. It may explain various radiological appearances of consecutive FC portions in the course of temporal bone development and also the absence of mastoid portion in our material, which may be due to its peripheral-most position [29].

The scans of the youngest fetuses in our study showed the primary places of ossification of the otic capsule that were similar to these in other studies [25, 27].

Comparing to the literature results, the lengths of the first and the second portions of FC were in all cases shorter than in adults, where they are reported as 2–6 mm for the labyrinthine and 8–12 mm for the tympanic portion [23]. However, detailed comparison of the adult FC and this of fetuses below 27 Hbd encountered technical complication, related mainly to inability of visualization of anterior wall of FC in our material. This observation implicates the fact that full ossification of FC is still not terminated in 27th week of gestation. Furthermore, the assessment of the measurements performed on fixed specimens and these taken from radiological studies creates confusion about their comparability. In our opinion, however, the use of micro-CT scans increases the accuracy of measurements and makes the landmarks more repeatable, regardless of the individual variability of the specimens.

Statistical analysis shows that the length of both portions of FC, labyrinthine (FC1) (*r* = 0.57, *p* < 0.05) and tympanic (FC2) (*r* = 0.55, *p* < 0.05), increases with age. This seems to be due to general elongation of the temporal bone in sagittal and horizontal planes [12].

The angle of the first curve in our material has similar value to this measured in adult specimens [23], which with no important differences in thickness of bony laminae (BCT, LSC) in our material and literature reports, may refer to the termination of the formation of the bony labyrinth, as suggested by other authors [18]. This fact may influence the morphology of the fallopian canal in the third trimester of pregnancy. Later ossification of anterior wall of FC in the region of the first curve (referring to the location of the geniculate ganglion) and tympanic portion may probably explain more common occurrence of bony dehiscences in these regions [9], which may favor accidental injuries of the facial nerve during otosurgical procedures in further life [15, 19]. According to the literature, dehiscences of FC are observed in up to 15 % of adult population [26] and show a positive correlation with an occurrence of facial nerve palsy in otogenic inflammatory diseases, such as acute and chronic suppurative otitis media [1] or cholesteatoma [10]. The statistical assessment shows a slight correlation of the value of A1-2 with age (*r* = 0.40, *p* < 0.05).

The average width of FC in adulthood ranges from 1.11 to 1.39 mm [24]. Comparing our results to the literature data, the average values for both portions of fetal FC were similar. We should emphasize the fact of this similarity despite different conditions for measurements of fetal FC, as the visualization of its walls cannot be performed as clearly as in adult FC due to incomplete ossification. The knowledge of eventual asymmetry of the width of FC may be of importance in the differential diagnostics of the lesions of the facial nerve of inflammatory and neoplastic origin [30]. The study of Sepahdari and Mong provides a statistical description of the asymmetry of this parameter for right and left side in adults. In our study, these values show a slight difference in the symmetry in FC1W (average 0.08; SD = 0.05; range 0.01–0.16 mm) and FC2W (average 0.07; SD = 0.07; range 0.00–0.19 mm).

The additional dimensions did not reveal any significant variants in fetal anatomy in relation to this reported in adults. The average length of the IAM in our material increases with age (*r* = 0.80, *p* < 0.05). Nevertheless, both distances BCT (*r* = 0.18, *p* > 0.05) and LSC (*r* = 0.30, *p* > 0.05) seem not to be influenced by age nor side.

## Conclusions

1. The level of ossification of consecutive portions of FC depends on age. Labyrinthine portion starts to ossify between 16th and 18th weeks of gestation and tympanic portion is fully ossified only after 20th week.
2. Labyrinthine and tympanic portion of FC and the IAM elongate with age, whereas the angle of the first curve of FC, the distances between the canal and the basal cochlear turn and the lateral semicircular canal remain stable and present no correlation with age.
3. Computed microtomographic examination is a useful method for assessment of FC in fetuses older than 18 Hbd as it gives a proper and reliable reference to other ossified bony structures of the temporal bone.
